# Management of eight labor and delivery patients dependent on buprenorphine (Subutex™): A retrospective chart review

**DOI:** 10.12688/f1000research.13350.2

**Published:** 2018-02-14

**Authors:** Solina Tith, Garinder Bining, Laurent Bollag

**Affiliations:** 1 Department of Anesthesia and Perioperative Care, University of California, San Francisco, CA, USA; 2 Department of Anesthesiology and Pain Medicine, University of Washington, Seattle, WA, USA

**Keywords:** Buprenorphine, Opioid tolerance, Pregnancy, Post Cesarean pain management, Regional anesthesia, Multimodal analgesia

## Abstract

**Background**: Opioid use during pregnancy is a growing concern in the United States. Buprenorphine has been recommended by “The American College of Obstetrics and Gynecology” as an alternative to methadone to decrease risks associated with the use of illicit opioids during pregnancy. The partial μ-opioid agonists’ unique pharmacology, including its long half time and high affinity to the μ-opioid receptor, complicates patient management in a highly kinetic, and often urgent field like obstetric anesthesia. We reviewed our management and outcomes in this medically complex population.

**Methods**: An Institutional Review Board (IRB) approved retrospective chart review was conducted of women admitted to the University of Washington Medical Center Labor and Delivery unit from July 2012 to November 2013 using buprenorphine. All deliveries, including intrauterine fetal demise, were included.

**Results**: Eight women were admitted during this period to our L&D floor on buprenorphine. All required peri-partum anesthetic management either for labor and/or cesarean delivery management. Analgesic management included dilaudid or fentanyl PCA and/or continued epidural infusion, and in one instance ketamine infusion, while the pre-admission buprenorphine regimen was continued. Five babies were viable, two women experienced intrauterine fetal death at 22 and 36 weeks gestational age (GSA), respectively, and one neonate died shortly after delivery due to a congenital diaphragmatic hernia.

**Conclusions**: This case series illuminates the medical complexity of parturients using buprenorphine. Different treatment modalities in the absence of evidence-based guidelines included additional opioid administration and continued epidural analgesia. The management of post-cesarean pain in patients on partial μ-opioid agonists remains complex and variable, and evidence-based guidelines could be useful for clinicians to direct care.

## Introduction

Opioid use during pregnancy is a growing concern in the United States. In a review of over 500,000 women, 76,742(15%) received at least one dose of an opioid during pregnancy and of these, 11,747 were dispensed opioids three or more times during pregnancy ^1^. The U.S. Food and Drug Administration (FDA) highlighted the need for further investigation regarding the risks of pain medicine use during pregnancy in a recent Drug Safety Communication in order to inform clinical practice ^2^. The FDA also emphasized that severe and persistent pain that is not effectively treated during pregnancy can result in maternal depression, anxiety, and high blood pressure ^2^.

The American College of Obstetrics and Gynecology (ACOG) released their opinion regarding opioid abuse, dependence, and addiction in pregnancy. They recommended buprenorphine as an alternative to methadone to decrease risks associated with the use of illicit opioids during pregnancy ^3^.

Buprenorphine (Subutex ^TM^) is a partial μ-opioid agonist and, at high doses, a weak κ-antagonist that is taken as a sublingual tablet ^4^. Suggested advantages of buprenorphine over methadone in pregnancy include less severe withdrawal symptoms, a lower risk of opioid overdose, fewer drug interactions, better ability to be treated on an outpatient basis without daily visits to a treatment program, less severe neonatal abstinence syndrome (NAS), and possibly less analgesic pain medications postpartum ^3, 5– 7^. On average, parturients taking buprenorphine did so for 131.6 (SD 98.7) days of their pregnancy ^1^.

At our institution, it is not uncommon for parturients to present for delivery while currently taking buprenorphine. Managing such patients, who generally have a long history of opioid abuse and addiction, is challenging, particularly when addressing post-cesarean pain management. Perfect anticipation of labor and delivery timing is not always possible. Buprenorphine’s long duration of action conflicts with the desired goal of tapering to a pure μ-opioid agonist prior to delivery.

This case series illustrates a range of presentations and multimodal treatments for patients taking buprenorphine on the labor and delivery ward, and explores the role of alternative pain management options, including epidural catheters, in these challenging cases.

## Materials and methods

After receiving Institutional Review Board (IRB) approval from the University of Washington Human Subjects Division (IRB #51693, Committee D), we performed a retrospective chart review to find parturients using buprenorphine or neonates, who received postnatal morphine to determine if their mother had been taking buprenorphine during pregnancy. We included all deliveries, including intrauterine fetal demise, from July 2012 to November 2013, on the University of Washington Medical Center Labor and Delivery unit.

## Results

There were 2521 deliveries from 7/1/2012 through 11/30/2013, of which, 152 (6%) received neonatal morphine. A chart review of the biological mothers of each of these neonates found that eight had been taking buprenorphine during pregnancy. Table 1 to Table 4 show the demographic, labor analgesia, Obstetric/Maternal outcome and neonatal outcome data of the eight patients identified. Individual cases are presented below.

**Table 1.** Demographic Data.

	1	2	3	4	5	6	7	8
**Age** **(years)**	37	27	34	28	21	22	35	34
**Gravity and** **Parity**	G3P1	G1PO	G5P2	G6P1	G4P3	G1PO	G4P2	G1P0
**Gestational Age** **(weeks and** **days)**	39 1/7	39 1/7	22 5/7	36 3/7	39	30 6/7	37 1/7	37 3/7
**Buprenorphine** **use upon L&D** **admission** **(mg/day)**	8	8	8	24	4	16	16	2
**Drug Use**	Heroin Benzos Cocaine	Heroin Opiates	Heroin	Benzos Opiates	Heroin	Heroin Meth THC	Opiates	Meth Opiates
**BMI** **(kg/m2)**	58	33.7	46	44.1	45	33.8	31.1	30

**Table 2.** Labor Analgesia Data.

Patient	1	2	3	4	5	6	7	8
**Labor Analgesia**	**N/A**	**CSE**	**CSE**	**N/A**	**CSE**	**N/A**	**CSE**	**CSE**
**VAS score range - while** **having labor analgesia**	**N/A**	0-3	0-7	**N/A**	0-6	**N/A**	0-4	0-4
**Epidural** **Top-Ups administered**	**N/A**	0	2	**N/A**	2	**N/A**	0	1

**Table 3.** Obstetric/Maternal Outcome Data.

Patient	1	2	3	4	5	6	7	8
**Mode of Delivery**	CS	CS	NSVD (D&C)	CS	NSVD	CS	NSVD	CS
**Indication**	Fetal Distress	Fetal Distress		Elective Repeat		Fetal Distress		Failure To Progress
**Anesthetic** **used for C/S if** **applicable**	GA after failed SPA and CSE	GA after failed labor analgesia conversion	N/A	GA per patient request	N/A	CSE	N/A	Labor analgesia converted
**Post Delivery** **Analgesia** **regimen**	LEP IV HM PO: APAP IBP OXY HM BUP	HM PCA PO: APAP IBP OXY BUP	PO: APAP IBP OXY SUB	FEN. PCA IV KETAMINEIV BENZO. PO: APAP IBP HM BUP	PO: APAP IBP OXY BUP	LEP HM PCA PO: APAP IBP HM BUP	PO: APAP IBP BUP	LEP HM PCA PO: APAP IBP OXY BUP
**24 hrs. post** **delivery** **VAS Pain score** **range**	0-5	0-8	0-7	6-8	0-4	2-8	2-7	3-9

•  24 hrs. Post Delivery Pain scores are presented as a range of lowest to highest reported pain score •  Respiratory Depression assessed by continuous pulse oximetry

**Table 4.** Neonatal Outcome Data.

Baby Patient	1	2	3	4	5	6	7	8
**APGAR scores at** **1 and 5 minutes**	4,8	4,7	IUFD	IUFD	5,6	8,6	8,9	8,9
**Cord Gas:** **Uterine Artery (UA)** **and** **Uterine Vein (UV)** **pH/pCO2/pO2/HCO3/** **Base Excess (BE)** **Base Deficit (BD)**	UA: 7.09/87/6/27/ BD:4.3 UV: 7.14/78/3/27/ BD 3.5	UA: 7.23/61/19/25/ BD: 3.6 UV: 7.25/56/30/24/ BD 4.0	N/A	N/A	UA: 7.26/61/21/27/ BD 2.2 UV: 7.31/48/30/24/ BD 2.7	UA: 7.33/58/21/30/ BE 3.6. UV: 7.39/47/33/28/ BE 2.8	UA 7.32/52/24/26/ BD 0.2 UV: 7.35/444/35/25/ BD 1.1.	UA 7.34/49/25/26/ BD 0.3 UV: 7.33/53/20/28/ BE 1.0
**Baby weight (grams)**	3414	3758	N/A	N/A	4036	1335	2533	3108
**Neonatal** **Interventions** **&** **NAS monitoring**	Routine newborn care, Photo Therapy. NAS monitoring negative	NICU admission due to Respiratory failure NAS monitoring positive			NICU admission due to CDH, severe pulmonary Hypoplasia. Palliative care and demise day 1	NICU admission due to VATER association w. subsequent corrective surgeries	Routine Newborn care NAS monitoring positive	Routine Newborn care NAS monitoring positive
**NAS Diagnosed**	No	Yes	N/A	N/A	No	No	Yes	Yes

**Table:** Legend for text and Tables 1–4.

APAP	= Tylenol
BD	= Base Deficit
BE	= Base Excess
Benzo	= Benzodiazepine
BUP	= Buprenophrine
CDH	= Congenital diaphragmatic hernia
CS	= Cesarean Section
CSE	= Combined Spinal Epidural Labor Analgesia
D&C	= Dilation and Curettage surgery
GA	= General Anesthesia
HM/Fent	= Hydromorphone or Fentanyl
IBU	= Ibuprofen
IOL	= induction of labor
IUFD	= Intra uterine fetal demise
IV	= Intravenous administration
LEP	= Lumbar Epidural Analgesia
Meth	= Methamphetamine
MVA	= motor vehicle accident
NAS	= Neonatal Abstinence Syndrome
NICU	= Neonatal Intensive care unit
NSVD	= Normal vaginal delivery
OXY	= Oxycodone
PCA	= patient-controlled analgesia
PEA	= pulseless electrical activity
PF	= preservative free
PMH	= past medical history
PO	= Per OS once cleared for orals
POD	= postoperative day
PRN	= as needed by patient
PROM	= premature rupture of membranes
s/p	= status post
SROM	= spontaneous rupture of membranes
SSEPS	= somatosensory evoked potential
SUB	= Suboxone
THC	= Cannabis
UA/V	= uterine Artery/Vein
VAS	= 11-point (0–10) Visual Analog Pain Score
VATER	= Vertebral defects, anal atresia, cardiac defects, tracheo-esophageal fistula, renal anomalies, and limb abnormalities

### Patient 1

37yo G3P1 at 39-1/7 weeks gestational age (GSA) who presented with vaginal bleeding and genital herpes. She had a history of polysubstance abuse and was started on buprenorphine (BUP) 8mg PO daily by an outside provider. On the day of admission (DOA), she had an urgent Cesarean section (CS) for possible abruption and fetal intolerance. Intraoperatively, a single shot spinal (SSS) with 100mcg of preservative-free (PF) morphine added to 12 mg of bupivacaine and 10mcg of fentanyl failed to provide adequate anesthesia. Subsequently, a combined spinal-epidural (CSE) using only 10mg of bupivacaine for the repeat spinal anesthesia was placed. Unfortunately, the patient complained of sharp incisional pain despite a negative Allis test to the T4 dermatome. She was then converted to a general anesthetic (GA).

Her post CS pain management included BUP at her admission dose, PO OXY (15mg Q3H), APAP, and IBP. The patient additionally received three doses of 0.4mg IV HM to treat breakthrough pain. Her epidural was continued for 24 hours postoperatively with 0.0625% bupivacaine at 10ml/H. At this point, the patient had successfully transitioned to a PO pain regimen and the epidural was removed. She was discharged on POD 6 (reportedly with inadequate pain control) on her pre-operative BUP dose along with a 10-day supply of HM 2–4 mg PO Q4hrs (120 pills).

### Patient 2

27yo G1P0 at 39-1/7 weeks GSA with a history of opioid dependence. She successfully completed an inpatient addiction treatment and was on BUP 8mg daily for one year. She was admitted for IOL in the setting of premature rupture of membranes (PROM). A CSE was placed on the DOA for labor analgesia and later an urgent CS was called for fetal distress. The epidural *in situ* was dosed for anesthesia in the operating room, but the patient reported a positive Allis test and consequently required a GA.

Post-operatively, the epidural was removed immediately since the epidural did not appear to provide operative anesthesia. She was not administered epidural morphine. Additional post-CS pain management included her pre-operative dose of BUP 8mg daily and a HM PCA; she was transitioned to PO OXY on POD 2. In addition, she did receive PO APAP and IBP throughout. On POD 1 she ambulated, met goals for symptom relief and was satisfied with her pain control. On POD 3, elevated blood pressures in the range of 120–150 mmHg systolic and 80–90 mmHg diastolic were measured and required treatment with furosemide and nifedipine.

She was discharged on POD 4 after a negative work-up of her hypertension. Discharge medications included a 7-day supply of OXY 5–15mg Q3H PRN (168 pills).

### Patient 3

34yo G5P2 at 22-5/7 weeks GSA with a history of bipolar disorder, morbid obesity, bicorneate uterus, and heroin abuse on buprenorphine-naloxone (Suboxone ^TM^) 8mg daily. Her obstetric history included two prior CS’s and an IUFD. She was admitted for IOL with an IUFD at 22 weeks GSA. A CSE was placed for labor analgesia on the DOA and provided adequate pain relief, but as her labor progressed, she required multiple top-up boluses.

After an uneventful NSVD the patient required a dilation and curettage for retained products. Her epidural catheter *in situ* was successfully used for the surgery, and removed afterwards. Epidural morphine was not administered.

Post-op pain management included PO OXY, APAP, and IBP and her home dose of Suboxone was re-initiated; the patient had discontinued it upon hospital admission.

On POD 1 the patient was diagnosed with a post-dural puncture headache and received an epidural blood patch with good effect. She required 10mg of PO OXY on 3 occasions during her hospital stay; however, at discharge on POD 1 she was not prescribed opioids.

### Patient 4

28yo G6P1 at 36 weeks GSA with cervical shortening, vaginal bleeding and pelvic pressure. She had a PMH significant for four years of BUP 8mg TID and alprazolam 1mg BID, opiate and benzo dependence, several 2nd trimester losses, and a CS at 40 weeks for 2nd stage arrest. She was diagnosed with an IUFD, and continued on her home dose of BUP and alprazolam while inpatient. The patient strongly desired GA for her CS.

Her post CS pain management included a ketamine infusion that was started intra-operatively at 8mg/H and continued post-op for 24H, a fentanyl PCA, PO APAP and IBP, as well as PRN IV lorazepam for anxiety. The patient’s PCA use of fentanyl included 4500mcg (1st 24H), 2600mcg (next 24H) and 3–6 mg IV lorazepam per day. On POD 2 the PCA was discontinued and PO HM was started. BUP was continued throughout her stay. The patient met goals for symptom relief and was satisfied with her pain control. She was discharged on POD 2 with a 10-day supply of HM 4mg PO Q6H (120 pills).

### Patient 5

21yo G4P3 at 39 weeks GSA with a body mass index (BMI) of 44, a history of previous low transverse CS, followed by successful vaginal birth after CS (VBAC) twice before. She had a history of heroin abuse and was on BUP 4mg/day. In this pregnancy the fetus had been diagnosed with CDH (congenital diaphragmatic hernia). The patient desired a trial of labor after C-section (TOLAC) and received a CSE for labor analgesia. She remained on her pre-admission dose of BUP throughout her hospital stay. Due to the fetus’ likely poor prognosis, medical staff decided that expediting birth of the fetus would be the safest course of action. After the rupture of membranes and labor augmentation she delivered on the DOA. Unfortunately, the infant died within hours of birth due to complications from CDH.

Her postpartum pain management included PO OXY, APAP and IBP, as well as her outpatient dose of BUP. Pain remained well controlled with this regimen. Her mood was somber and she was grieving appropriately. Postpartum complications included elevated blood pressures without features of pre-eclampsia on post-partum day 1 (PPD). With well controlled pain and appropriate functional status, she was discharged three days after delivery. By the end of the hospital stay, she only required scheduled PO APAP and IBP for pain; she was discharged with no additional short acting opioids.

### Patient 6

22yo G1PO at 30-6/7 weeks GSA who presented with preterm PROM. The pregnancy was complicated by heroin and methamphetamine abuse during the first trimester. After admission to the antepartum unit, her home dose of daily BUP 16mg for the remainder of her pregnancy was ordered. On the third day of the hospitalization, prolonged fetal decelerations prompted an urgent CS. A routine CSE was placed for CS anesthesia, and the surgery proceeded uneventfully. The spinal dose included bupivacaine 12.5mg, PF morphine 100mcg, and fentanyl 10mcg. Ketorolac 30mg IV was administered at the end of the case per routine protocol.

Her post CS pain management included an epidural infusion of bupivacaine 0.0625% at 10cc/H, a HM PCA, PO APAP and IBP and her daily home dose BUP. The patient’s pain was well-controlled and she was fully satisfied with pain management. After successful transition to PO HM the epidural was removed. The patient remained satisfied with her pain relief and was discharged on POD 2 with 36 tabs of 2mg HM.

### Patient 7

35yo G4P2 at 37-1/7 weeks GSA who presented for IOL in the setting of term IUFD in a previous pregnancy. She had a history of opioid dependence following an injury in the military requiring multiple reconstructive knee surgeries. She was placed on BUP 16mg daily for the remainder of her pregnancy and received this also throughout her hospital stay. During her IOL she received a CSE for labor analgesia, followed by an uncomplicated vaginal delivery 2 days after admission.

Her postpartum pain management included PO APAP and IBP and her daily home dose BUP; epidural was removed after delivery. She did not require additional PO opioids during her hospital stay and was discharged without any additional short acting opioids on POD 2.

### Patient 8

34yo G1PO at 37-3/7 weeks GSA who presented with SROM. She had a history of opioid dependence following an MVA, in addition to current methamphetamine use. Her PMH was also significant for a congenital ventricular septal defect s/p surgery at age 1yo, with secondary pulmonary stenosis and a dilated right ventricle with mild dysfunction. In addition, the patient had a complex partial seizure disorder, tobacco use, and poor compliance with pregnancy care. She had been on BUP 2mg BID throughout the pregnancy, which was continued during her L&D stay. She received a CSE on the DOA for labor analgesia and required a CS for second stage arrest a day later; the epidural catheter *in situ* was successfully converted to provide anesthesia. She was not given epidural morphine.

Her post CS pain management included a HM PCA, an epidural infusion (0.1% bupivacaine at 8cc/H), PO APAP, IBP and her home dose of BUP. On POD 2 the epidural infusion was discontinued. On POD 3 she was transitioned to PO OXY and the PCA stopped. She was counseled not to use amphetamines while breastfeeding. On POD 5 she was discharged to home with 30 tabs of 5mg OXY, with the plan of continuing BUP in the outpatient setting.

## Discussion

This retrospective chart review shows the heterogeneity and complexity of peripartum pain management in patients on buprenorphine (Subutex ^TM^) therapy ( Table 3). Neuraxial techniques, namely continued utilization of epidural catheters placed for labor and/or the cesarean delivery was the most common post-operative analgesic method used or offered to patients. Despite the use of chronic opioids, our routine dilute epidural solution (1/16% of Bupivacaine +2mcg Fentanyl /ml), after the spinal dose of the CSE wore off, provided satisfying labor analgesia. While lumbar epidural analgesia provides effective post cesarean analgesia ^8– 10^, the associated motor block hinders mobilization, often necessitating that epidural infusions be stopped on POD 2, in comparison to other surgical populations where epidural analgesia can be used longer ^11^.

In addition to our standard post-CS multimodal analgesic regimen, which includes neuraxial opioids, PO APAP, NSAIDs, and OXY, IV ketamine is utilized mainly as a rescue medication for intractable pain ( Table 3). One patient with a non-viable fetus received a low dose (8mg/H) ketamine infusion post-operatively. NMDA receptor antagonist infusions are rarely used on our L&D floor, in part due to uncertainty of fetal central nervous system effects ^12, 13^. Similarly, gabapentinoids are reserved for cases where the pain management is complex, due to unclear fetal effects and reported maternal sedation ^14^. None of our reported cases received this class of drug. Most patients received additional IV opioids after their CS’s. Fentanyl was used in one case, while HM was used in four cases ( Table 3). The most effective opioid in the setting of concurrent BUP remains unclear, some suggest using morphine ^15^. The particular strong µ-opioid receptor affinity of BUP, however, complicates the titration of commonly used pure agonists for pain management. To allow for better titration, some suggest the use of shorter acting opioids, like fentanyl, which patient 5 received, in line with our acute pain service recommendations. Ideally, a regional anesthetic technique combined with a PCA and possible use of an adjunct analgesic like ketamine and or gabapentin is used for post cesarean analgesia.

In many of the cases we described, transitioning patients from IV to PO opioid pain medication proved challenging and often required a prolonged hospital stay. OXY is our routine PO opioid and we found it to be effective in six cases; two women preferred PO HM. A retrospective study that matched patients treated with BUP to control patients found that patients maintained on BUP have similar intrapartum pain and analgesic needs during labor,yet experience more postpartum pain and use more opioid analgesia following cesarean delivery ^16^. Theoretically, adding opioids to the local anesthetic epidural infusion for post-operative pain management, compared to an IV PCA system, may reduce maternal plasma levels and subsequent fetal opioid exposure. However, we found this not feasible in our teaching institution setting.

All patients in our series were continued on their home dose of BUP throughout hospitalization ( Table 1). One key consideration is whether patients should be tapered off BUP prior to delivery when operative techniques may be necessary. One case report described a woman who tapered from 24mg of BUP starting at 14 weeks GSA ^17^. The patient demonstrated increased withdrawal symptoms and her fetus showed signs of distress. The woman was re-initiated on BUP and delivered without complication. Further study is needed to investigate the appropriate tapering methods in this population, and each patient’s medical history and psychosocial background must be carefully evaluated. The potential risks of tapering, including autonomic effects and withdrawal symptoms, to both the mother and fetus may not be justified in many cases. The current evidence continues to support the relative safety of BUP; one study found that women who taper their BUP by more than 50% during pregnancy did not have significantly different neonatal outcomes compared to women who remained on the same dose. ^18^. Also, fewer term NAS infants require drug treatment if exposed to BUP compared to methadone. ^19^. Yet, three out of six newborn were diagnosed with NAS in this series. The correlation of NAS with maternal opioid dependence is well known and should guide post-natal infant monitoring, regardless of the opioid used.

Three women in our series relapsed into pre-pregnancy habits of opioid abuse. One woman unfortunately overdosed and a subsequent urine sample was positive for oxycodone and its metabolites. This emphasizes the importance of post-hospital care and follow-ups in this high-risk population. To this end, the University of Washington operates a perioperative pain clinic staffed with specialized physicians and pharmacists that follow-up with high risk patients. In this setting, opioid weaning can be professionally supported until the regimen is deemed manageable by the primary provider. Utilization of this service is patient dependent, and social disarray is a risk factor for poor compliance.

The management of post-cesarean pain in patients on partial μ-opioid agonists remains complex and variable, and evidence-based guidelines could be useful for clinicians to direct care. Pre-existing protocols, customized to provide flexibility, could be extremely valuable in a setting that is by its very nature, highly kinetic and often urgent. It is crucial that health care providers dealing with these complicated patients are aware of possible options that offer safe treatment.

## Data availability

All gathered data was taken directly from patient files, de-identified and entered in the tables presented.

## Ethics and consent

Approval for the study was obtained from the Institutional Review Board (IRB) of the University of Washington Human Subjects Division (IRB #51693, Committee D). For this study a waiver of consent from patients was obtained from the IRB.
