# Supplementary material for: Management of eight labor and delivery patients dependent on buprenorphine (Subutex™): A retrospective chart review
Source: F1000Res. 2018 Feb 14;7:7. Originally published 2018 Jan 3. [Version 2] doi: 10.12688/f1000research.13350.2 (PMC5820592; doi:10.12688/f1000research.13350.2)
Supplement: Peer review history

# Peer review: Referee response for version 2

**DOI:** 10.5256/f1000research.15197.r30882

**Published:** 2018-02-19

**Authors:** Allison J. Lee

**Affiliations:** 1 Department of Anesthesiology, Columbia University Medical Center , New York City, NY, USA

General Comments:

 The authors present a very interesting retrospective report of the peripartum anesthetic management of 8 obstetric patients who had been receiving buprenorphine treatment during pregnancy. The issue is very topical and of great current concern for clinicians. The case reports are a valuable contribution to the medical literature. As the authors raise in the discussion, evidence-based protocols to direct care in this clinical scenario are warranted. The unique challenges of managing obstetric patients taking buprenorphine is not well described, however, citing more recent references e.g. Jones et al. ^1^ or the recent case series by Leighton and Crock ^2^ would be recommended.

 Although the management of the individual cases were outlined clearly, a description of the reasons for the challenges to analgesia management presented by buprenorphine maintenance in general, and specifically for the obstetric patient could have been better described. For example, it would have been useful to explain the potential for reduced efficacy of additional opioids where buprenorphine is continued, because of the high-affinity mu-receptor binding of buprenorphine, as well as opioid antagonism by naloxone in suboxone users. While there is limited evidence about best practices for management, a few sentences discussing the known pros and cons of discontinuing vs. continuing the drug(s), including risk of withdrawal, but potentially improved analgesia could have been more clearly laid out. Most cases were patients who presented unexpectedly, but patients with anticipated admissions, may have been managed differently, and that issue might have been mentioned.

 The authors are advised to adhere to precise medical terminology. There were several instances of imprecise medical language/colloquialisms throughout the text. There is repeated reference to a negative or positive “Allis test”, presumably meant to refer the obstetric surgeon’s verification of the adequacy of sensory blockade by clamping the skin with Allis tissue forceps. The authors are advised to use universally understood medical terminology. The term could be confused with the “Allis sign” or “Galeazzi test” used by orthopedic surgeons. There were several references to a “catheter” or “patient” being converted to a type of anesthetic, where more precisely, the epidural catheter in place was successfully used to provide a surgical anesthetic, or a decision was made to induce general anesthesia. Finally, the plethora of abbreviations were often difficult to follow, sometimes inconsistently used, and mostly unnecessary with respect to readability.

 Specific Comments:

 Introduction - The authors mention that it is “not uncommon” at their institution for parturients to present for delivery while currently taking buprenorphine. It would be useful to provide an estimate of what proportion of women do present with this issue, or with what frequency this issue is encountered in their practice.

 Methods - Some additional details of the methodology of chart review would be useful. The reader could be informed, for example, whether there are electronic records at that institution and if so, which search terms were used, and so on.

 Results - The authors mention the number of deliveries and mention that the “deliveries” received neonatal morphine, which is incorrect grammatically.

 Discussion - A clearer summary statement outlining a prudent approach to the opioid dependent patient, in the absence of an established protocol would be helpful information. This could include a multimodal approach utilizing neuraxial/regional blockade, use of non-opioid analgesic agents, and so on. Consultation with acute pain experts could have been considered in the most difficult cases, and the ideal follow-up care could have been mentioned.  Some of these issues were indeed raised, but in a less organized fashion.

 Tables - The units of measure for elements such as the base excess and base deficit should be included. The term “Apgar” is a name and the term should not be in all capital letters. The labels would benefit from revisions in multiple areas. For example, “Indication” in Table 3, would be clearer if the label were “Indication for cesarean delivery”.

I have read this submission. I believe that I have an appropriate level of expertise to confirm that it is of an acceptable scientific standard.

## References

[1] JonesHEDeppenKHudakMLLeffertLMcClellandCSahinLStarerJTerplanMThorpJMWalshJCreangaAA: Clinical care for opioid-using pregnant and postpartum women: the role of obstetric providers. *Am J Obstet Gynecol*.2014;210(4) : 10.1016/j.ajog.2013.10.010 302-310 10.1016/j.ajog.2013.10.010 24120973PMC7213596

[2] LeightonBLCrockLW: Case Series of Successful Postoperative Pain Management in Buprenorphine Maintenance Therapy Patients. *Anesth Analg*.125(5) : 10.1213/ANE.0000000000002498 1779-1783 10.1213/ANE.0000000000002498 29049122PMC5642324

# Peer review: Referee response for version 1

**DOI:** 10.5256/f1000research.14490.r29456

**Published:** 2018-02-01

**Authors:** Emily J. Baird

**Affiliations:** 1 Department of Anesthesiology and Perioperative Medicine, Oregon Health & Science University, Portland, OR, USA

Tith et al. highlight the complexity of the analgesic management of labor and delivery in parturients receiving buprenorphine. The retrospective chart review details the peripartum course of 8 women on buprenorphine maintenance. Given the heterogeneity in patient demographics, buprenorphine dose, analgesic regimen, mode of delivery, and neonatal outcomes, it is difficult to extract meaningful conclusions. The vast disparateness of the peripartum management of parturients on buprenorphine vividly demonstrates the need for evidence-based practice guidelines.

 Although the details of the individual patient’s peripartum course are interesting, the comprehensiveness of each description is distracting. Since the focus of the review is the analgesic management of labor and delivery on patients receiving buprenorphine, consider omitting extraneous maternal and neonatal details. The patients’ descriptions should conclude with discharge. Details such as “two weeks after delivery, patient was found pulseless…,” “patient stayed with her baby at the local children’s hospital…,” and “in the following days, she returned to clinic requesting opioids due to breast pain” detract from the intention of the review. Similarly, the specifics of the neonate’s postdelivery course (i.e. diagnosis of imperforate anus) are irrelevant. Concise reconstruction of the results section will highlight the focus of this review.

 Tables 1-4 are not referred to in the text. Without further explanation of the tables in the text, it is unclear what information the table is intended to convey.

 Table 2 (Labor Analgesia Data) is a bit misleading. For patient 1, the table indicates the patient had a CSE for labor that required no “top-ups” and resulted in a VAS score of zero. According to the results section, patient 1 had a failed single shot spinal, followed by a CSE, and ultimately needed a general anesthetic for cesarean delivery. This seems to suggest that the patient never received labor analgesia but rather the CSE was placed for surgical anesthesia.

 The absences of a comprehensive legend for Table 3 makes it challenging to interpret. Twelve abbreviations are used in Table 3 which are not defined until the following page. Consider including a key to the abbreviations in the table legend. In addition, since respiratory depression did not occur in any parturient, consider removing it from the table.

 The discussion section would be more meaningful if it offered some interpretation of the data rather than summarizing the results presented in the previous section. Specifically, why did 3 of the 5 women undergoing cesarean delivery have a general anesthetic? Based on the limited experience, what is the optimal labor analgesia regimen? Post vaginal delivery pain regimen? Post-operative regimen? Neonatal implications of intrauterine exposure to buprenorphine?

 Tith et al.’s retrospective review of the periparturm course of parturients dependent on buprenorphine illustrates the heterogeneity of analgesic regimens for labor and delivery. The review highlights the need for research to help develop protocols and standards.

I have read this submission. I believe that I have an appropriate level of expertise to confirm that it is of an acceptable scientific standard.
